# Molecular basis for cellular retinoic acid-binding protein 1 in modulating CaMKII activation

**DOI:** 10.3389/fmolb.2023.1268843

**Published:** 2023-09-26

**Authors:** Jennifer Nhieu, Michelle C. Miller, Thomas A. Lerdall, Kevin H. Mayo, Li-Na Wei

**Affiliations:** ^1^ Department of Pharmacology, University of Minnesota Medical School, Minneapolis, MN, United States; ^2^ Department of Biochemistry, Molecular Biology and Biophysics, University of Minnesota, Minneapolis, MN, United States

**Keywords:** CRABP1, CaMKII, NMR, molecular interaction, kinase activation, beta-barrel protein

## Abstract

**Introduction:** Cellular retinoic acid (RA)-binding protein 1 (CRABP1) is a highly conserved protein comprised of an anti-parallel, beta-barrel, and a helix-turn-helix segment outside this barrel. Functionally, CRABP1 is thought to bind and sequester cytosolic RA. Recently, CRABP1 has been established as a major mediator of rapid, non-genomic activity of RA in the cytosol, referred to as “non-canonical” activity. Previously, we have reported that CRABP1 interacts with and dampens the activation of calcium-calmodulin (Ca^2+^-CaM)-dependent kinase 2 (CaMKII), a major effector of Ca^2+^ signaling. Through biophysical, molecular, and cellular assays, we, herein, elucidate the molecular and structural mechanisms underlying the action of CRABP1 in dampening CaMKII activation.

**Results:** We identify an interaction surface on CRABP1 for CaMKII binding, located on the beta-sheet surface of the barrel, and an allosteric region within the helix segment outside the barrel, where both are important for interacting with CaMKII. Molecular studies reveal that CRABP1 preferentially associates with the inactive form of CaMKII, thereby dampening CaMKII activation. Alanine mutagenesis of residues implicated in the CaMKII interaction results in either a loss of this preference or a shift of CRABP1 from associating with the inactive CaMKII to associating with the active CaMKII, which corresponds to changes in CRABP1’s effect in modulating CaMKII activation.

**Conclusions:** This is the first study to elucidate the molecular and structural basis for CRABP1’s function in modulating CaMKII activation. These results further shed insights into CRABP1’s functional involvement in multiple signaling pathways, as well as its extremely high sequence conservation across species and over evolution.

## 1 Introduction

Cellular retinoic acid-binding protein 1 (CRABP1) is a highly conserved protein that is believed to function by binding and sequestering cytosolic retinoic acid (RA), which is important in controlling cellular RA concentration (Napoli, 2016; Napoli, 2017). This view centers on the well-known genomic activities of RA, mediated by nuclear RA receptors (RARs) that regulate gene expression, generally referred to as the canonical activities of RA (Wei, 2003; Cunningham and Duester, 2015). An increasing number of studies have identified rapid cytosolic (without involving nuclear RARs) activities of RA, which could modulate cytosolic signaling pathways as demonstrated in multiple cell types, including adipocytes (Lin, 2020a; Wei et al., 2022), cardiomyocytes (Park et al., 2018; Park et al., 2019), stem cells (Persaud et al., 2013; Lin et al., 2017), and neurons (Lin, 2020b; Lin et al., 2022). Recently, it has been established that CRABP1 acts as a direct mediator of these rapid cytosolic activities of RA in a cell context-dependent manner, referred to as non-canonical RA activities reviewed in Nagpal and Wei (2019).

In embryonic stem cells, adipocytes, and neural stem cells that are extremely sensitive to growth factor-elicited signaling pathways such as the mitogen-activated protein kinase (MAPK) pathway (Zhang et al., 2002; Wei, 2013), CRABP1 is important for stem cell proliferation (Persaud et al., 2013; Lin et al., 2017), exosome secretion (Lin et al., 2021), and adipokine production (Wei et al., 2022). The MAPK pathway is initiated by the membrane-bound Ras GTPase, which serves as a molecular switch to activate the Raf-MEK-ERK kinase cascade upon growth factor stimulation (Cargnello and Roux, 2011). In this pathway, CRABP1 competes with Ras for direct interaction with Raf kinase, ultimately resulting in dampened ERK activation in cells highly expressing CRABP1 (Nagpal and Wei, 2019; Wook Park et al., 2019). Using nuclear magnetic resonance (NMR) spectroscopy, we have revealed a Raf-interacting surface area on the beta-sheet face of CRABP1, providing molecular insight and a potential structural basis of CRABP1’s function in negatively regulating MAPK signaling (Wook Park et al., 2019). In this context, CRABP1 has been found to be a negative regulator acting to “dampen” or “safeguard” the activation of the MAPK signaling pathway that is crucial to growth factor-sensitive cells.

We have recently uncovered that CRABP1 can also elicit specific non-canonical activity to negatively modulate Ca^2+^-calmodulin dependent kinase 2 (CaMKII) signaling (Park et al., 2018; Lin et al., 2022). CaMKII is a serine/threonine kinase and a major mediator of Ca^2+^ signaling for essential functions of excitable cells such as cardiomyocytes and neurons. CaMKII exists in four major isoforms: alpha, beta, delta, and gamma, with alpha and beta predominantly expressed in the CNS and delta expressed in the cardiac tissues (Erickson, 2014; Zalcman et al., 2018). In cardiomyocytes, CaMKII plays a crucial role in the coordination of excitation–contraction coupling required for the mechanical activity of cardiac tissue (Erickson, 2014). In neurons, CaMKII is best known for its critical role in synaptic transmission, essential for most neuronal functions, including motor activity, learning, and memory (Lisman et al., 2002; Lisman et al., 2012). As a major mediator of Ca^2+^ signaling in excitable cells, CaMKII is directly activated through elevated cytosolic Ca^2+^ which binds calmodulin (CaM). The Ca^2+^-bound CaM then directly binds CaMKII, freeing the kinase from its autoinhibited, inactive state to target/phosphorylate-specific downstream substrates necessary for the functions of cardiomyocytes and neurons (Stratton et al., 2013; Bhattacharyya et al., 2020). Critical to disease processes, overactivation of CaMKII activity in both cardiomyocytes and neurons is a well-documented pathophysiological driver of cardiac and neurological diseases (Ashpole and Hudmon, 2011; Robison, 2014; Zhang, 2017). However, effective modulation or control of CaMKII activation in order to manage these diseases remains a challenge (Pellicena and Schulman, 2014). To this end, both cardiomyocytes and motor neurons highly express CRABP1, and CRABP1 can negatively regulate CaMKII activation (Park et al., 2018; Lin et al., 2022).

In mice, upon beta-adrenergic assault and subsequent CaMKII overactivation, CRABP1 protects against cardiac damage by dampening CaMKII overactivation in cardiomyocytes (Park et al., 2018). In mouse motor neurons (MNs), CRABP1 dampens CaMKII overactivation, thereby maintaining healthy production of agrin in MNs, a proteoglycan essential for the maintenance of neuromuscular junction health (Li et al., 2018; Lin et al., 2022). Recently, we have further reported that RA and CRABP1 ligands, C4 and C32, can protect against excitotoxic assault in MNs grown in cultures (Nhieu et al., 2023). As such, understanding how CRABP1 modulates CaMKII activation can be potentially helpful in designing therapeutic strategies for cardiac and neurological diseases.

In this study, through biochemical and molecular studies, we have uncovered a molecular/structural basis for CRABP1’s action in modulating CaMKII activation. NMR data reveal a potential interaction surface on CRABP1 for interacting with the regulatory (R) domain of CaMKII. Molecular studies show that CRABP1 preferentially interacts with the inactive CaMKII, which provides a molecular basis for the “dampening” effect of CRABP1 with regard to the activation of CaMKII. Data of site-specific mutations of the R domain-interacting surface of CRABP1 confirm the stringent requirement for CRABP1 to maintain this R-interacting surface on the beta-sheet barrel, as well as the alpha-helix segment outside the barrel. We also discuss the molecular and structural basis underlying the highly conserved nature of CRABP1 with regard to its signaling pathway-specificity and primary sequence conservation throughout evolution.

## 2 Results

### 2.1 Structural regions on CRABP1 contribute to its interaction with CaMKII and modulation of CaMKII activation

**FIGURE 1:**
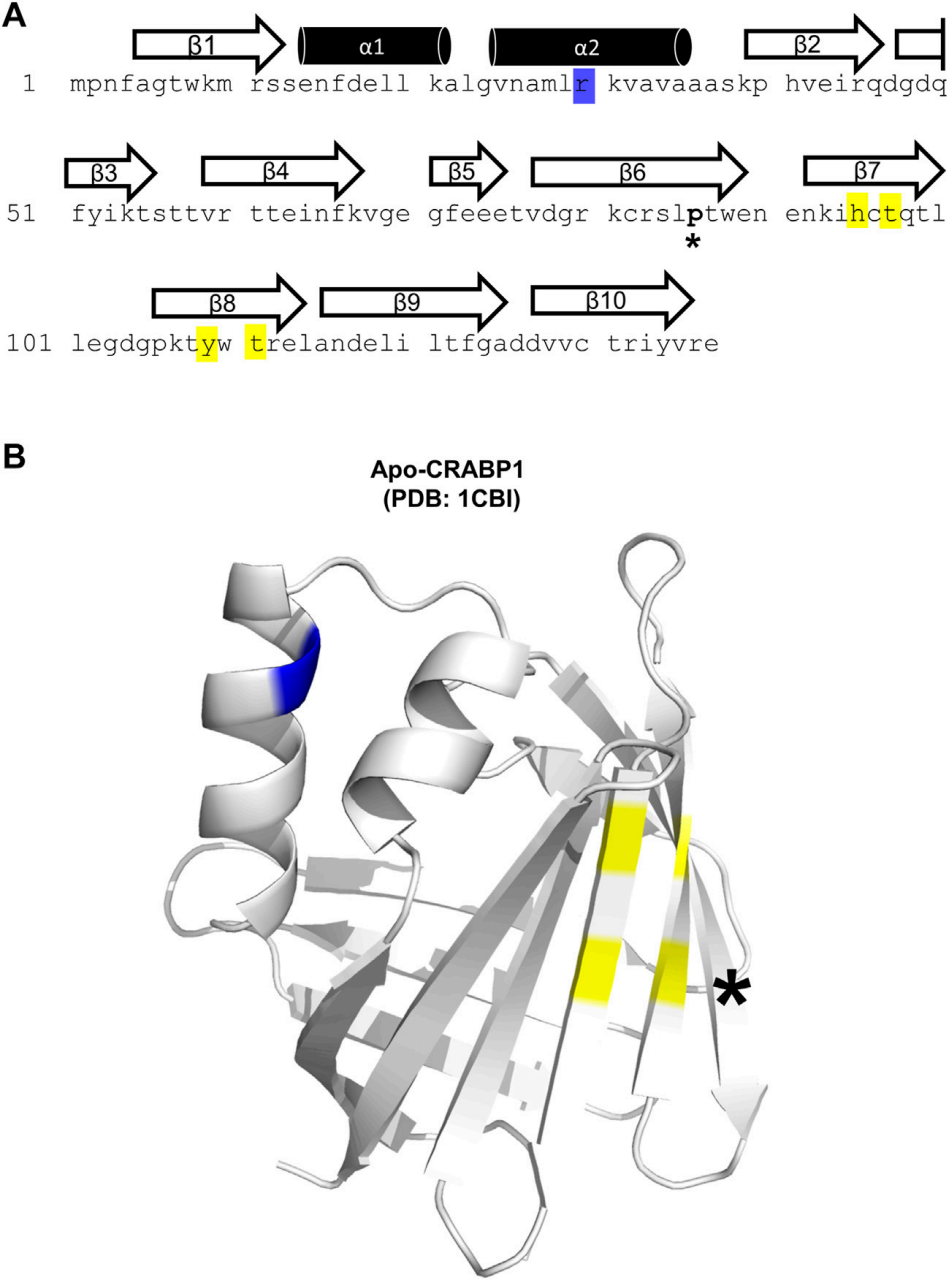
Structural details of the CRABP1 protein. **(A)** CRABP1 amino acid sequence from *Mus musculus* (UniProt ID P62965). Secondary structures are superimposed above their relevant sequence. Yellow and blue highlights indicate the residues identified in this study relevant to CaMKII modulation. The asterisk (*) indicates the only non-conserved residue, proline 86, across CRABP1 mammalian orthologs. **(B)** Crystal structure of apo-CRABP1 (ligand-free, PDB1CBI) with the residues of interest mapped to the helix-turn-helix motif (blue) and beta-sheet face (yellow). The non-conserved proline 86, located outside the regions of interest, is marked by an asterisk (*). The crystal structure image was generated using PyMOL software.

The CRABP1 protein is a member of the intracellular lipid-binding protein (iLBP) superfamily, characterized by an anti-parallel beta-barrel tertiary structure that forms the ligand-binding pocket (Banaszak et al., 1994; Thompson et al., 1995; Schaap et al., 2002). This CRABP1 barrel consists of 10 beta-strands and is linked to a helix-turn-helix motif (Thompson et al., 1995). Figure 1A shows the highly conserved (>99%) sequence of the *Mus musculus* CRABP1 isoform (Uniprot ID P62965), with the relevant secondary structures superimposed over the corresponding amino acid sequence. The only non-conserved residue is proline 86, which exists as an alanine in the human and bovine orthologs (Figure 1A, asterisk) (Nhieu et al., 2022). In this study, we examine residues within helix 2 (blue highlight) and beta strands 7 and 8 (yellow highlights) that all contribute to its activity in interacting with, and regulating, CaMKII (Figure 1A). On the crystal structure of CRABP1 (apo form, PDB: 1CBI (Thompson et al., 1995) are located these residues, either in the helix-turn-helix motif (blue) or the beta-sheet surface of the barrel (yellow) (Figure 1B). Interestingly, the only one non-conserved residue, proline 86 (Figure 1B, black asterisk), is located outside of both regions (helix 2 and beta strands 7 and 8), suggesting that this non-conserved residue is probably not critical for CRABP1’s function in this context.

### 2.2 Characterization of a CaMK-R peptide for CRABP1–CaMKII interaction studies

**FIGURE 2:**
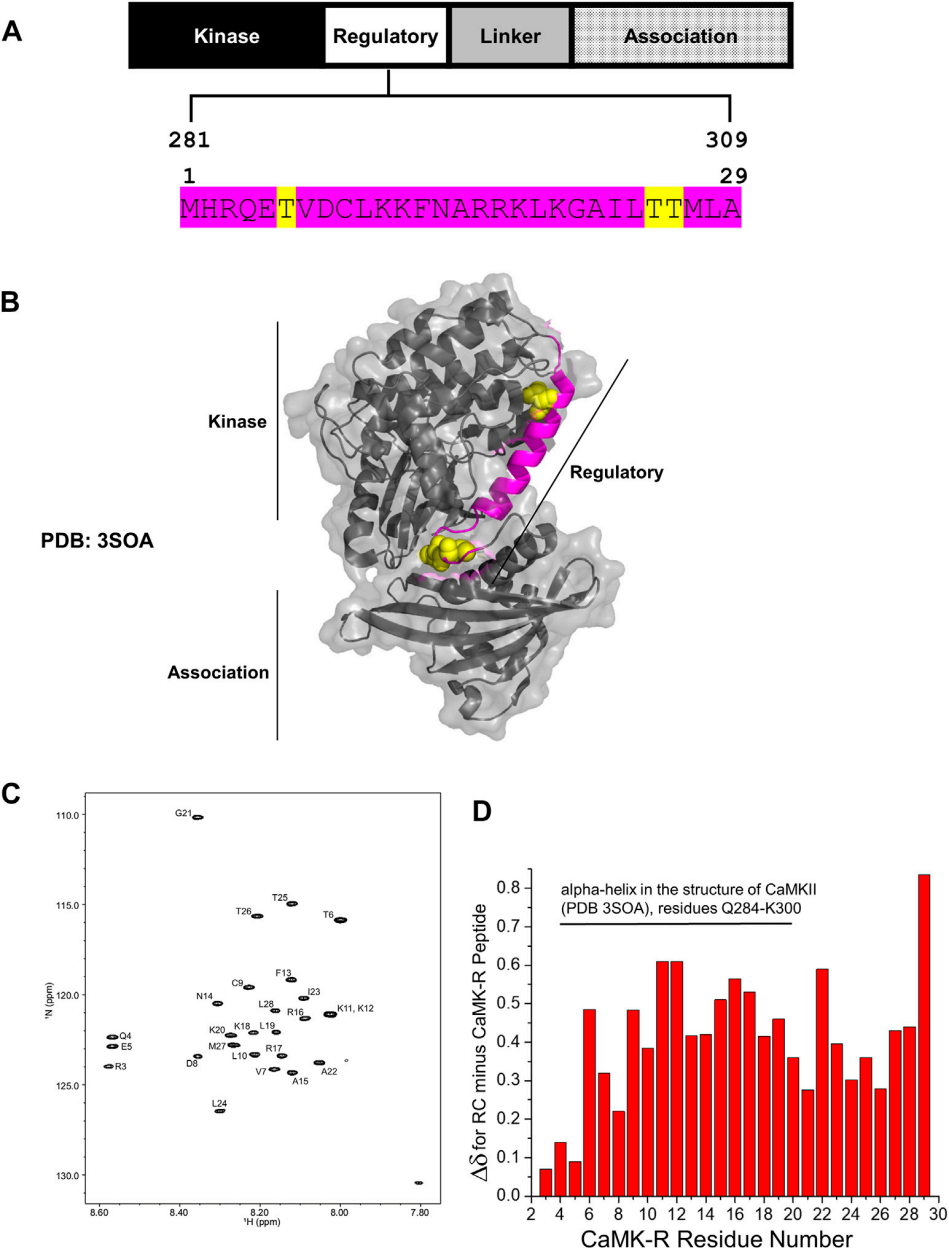
Structural details of the CaMK-R peptide. **(A)** The 29-residue CaMK-R peptide sequence (magenta) mapped to residues 281–309 of the regulatory segment of full-length CaMKII and its functional domains. Major regulatory residues threonine 286/7, 305, and 306 are highlighted in yellow. The residue numbering of CaMKII is based on the *Mus musculus* CaMKII alpha isoform (Uniprot ID P11798). **(B)** The 29-residue CaMK-R peptide sequence (magenta) mapped to residues 281–309 of the full-length crystal structure of autoinhibited CaMKII (PDB 3SOA). Regions of the kinase, regulatory, and association domains within the CaMKII crystal structure are indicated by the black solid lines. Major regulatory residues threonine 286/7, 305, and 306 are displayed as yellow space-filling spheres. **(C)** HSQC spectra of the CaMK-R peptide. Spectra were generated using Bruker TopSpin Software. **(D)** Chemical shift changes (Δδ) for the difference between random coil (RC) values and the CaMK-R peptide values plotted against CaMK-R residue number. CaMK-R residues 4–20 map to residues 284–300 of full-length CaMKII. The plot was generated using Origin Software.

CaMKII is a serine/threonine kinase comprising three major functional domains—the kinase domain, regulatory (R) segment, variable linker region, and association domain (Bhattacharyya et al., 2020) (Figure 2A). Within the R segment reside three key threonine (Thr) residues: Thr286/7 (depending on isoform), 305, and 306 (Figure 2A, yellow highlight) (Bhattacharyya et al., 2020). The binding of calcium–calmodulin (Ca^2+^–CaM) to the R segment frees CaMKII from its autoinhibited basal state and exposes Thr286/7 to autophosphorylation, thereby activating CaMKII. Autophosphorylation of Thr305 and 306 blocks Ca^2+^–CaM binding, preventing further activation of the kinase by Ca^2+^–CaM (Bhattacharyya et al., 2020). Thus, phosphorylation of Thr286/7 is a key marker for activated CaMKII. In this study, activated CaMKII and CaMKII phosphorylated at Thr286/7 (pCaMKII T286/7) will be considered synonymous. It should be noted that all domains, except for the variable linker region, are highly conserved among all the CaMKII isoforms and across species, indicating the functional conservation for each of these domains regardless of the CaMKII isoform or homolog (Stratton et al., 2013; Bhattacharyya et al., 2020).

In our previous studies, we have determined that CRABP1 directly interacts with CaMKII on its three functional domains—kinase domain, R segment, and association/oligomerization domain (Park et al., 2018; Lin et al., 2022). Furthermore, with regard to CaMKII interaction, CRABP1 competes with calmodulin (Park et al., 2018) which binds directly on the R segment of CaMKII and is the major positive regulator of CaMKII activation (Bhattacharyya et al., 2020). Given the “modulatory” nature of CRABP1’s action in CaMKII activation, these current studies focus on characterizing CRABP1 interaction with the R-segment of CaMKII. Furthermore, NMR spectroscopy approaches were used in order to understand the molecular interactions of CRABP1 with the R-segment in solution. We first synthesized a 29-residue peptide (Figure 2A, magenta sequence) derived from residues 281–309 of the R segment of *Mus musculus* CaMKII alpha (Uniprot ID P62965), referred to as “CaMK-R” in the following text. This CaMK-R was used *in lieu* of the full-length protein due to the mass/size limitations of using NMR (Puthenveetil and Vinogradova, 2019). For in-cell functional studies (active vs. inactive CaMKII), we exploited an expression construct of the CaMKII beta isoform from *Rattus norvegicus* (Addgene #21227). CaMKII itself endogenously forms protein complexes comprising 12–14 subunits, with each subunit having a molecular weight (MW) of ∼50–60 kDa. A single subunit comprises the three functional domains. Protein complex formation of the CaMKII multimer is facilitated by interactions between the association domains of each subunit (Stratton et al., 2013; Bhattacharyya et al., 2020). Figure 2B shows a PyMOL structure of an individual, full-length CaMKII subunit in its basal autoinhibited form [PDB: 3SOA (Chao et al., 2011)]. Residues that comprise CaMK-R are highlighted in magenta, along with Thr286/7, 305, and 306 as van der Waals spheres in yellow.

To ensure that the CaMK-R peptide was appropriate for the CRABP1–CaMKII interaction studies using NMR, we initially used the PEP-FOLD peptide structure prediction server (Lamiable et al., 2016) to predict the secondary structure of CaMK-R. The five top-ranked models indicate that CaMK-R most probably has an alpha-helical structure (Supplementary Figure S1). To assess whether this proposed helical structure of CaMK-R can properly form in solution, we performed NMR experiments (^1^H TOCSY and NOESY) to make sequence-specific resonance assignments (Supplementary Figure S2). For additional information on the chemical shifts of CaMK-R, we acquired a natural abundance ^15^N-^1^H HSQC spectrum of CaMK-R, with assigned peaks as labeled (Figure 2C). Calculation of the difference between random coil (RC) chemical shifts and observed chemical shifts for each amino acid (Δδ = δ_RC—_δ_observed_) (Wuthrich, 1991) can provide information on the presence and type of the secondary structure (Wishart et al., 1991). A series of positive Δδ values (0.1 ppm above RC values) suggest the presence of an alpha-helical structure, whereas a series of negative Δδ values suggest the presence of a beta-sheet structure. Positive Δδ values calculated for CaMK-R residues support the idea that N-terminal residues 4–20 (residues 284–300 in full-length CaMKII) likely have an alpha-helical structure (Figure 2D). Given the relatively small change in Δδ between observed and RC chemical shifts, this helical “structure” in CaMK-R is actually transient, occurring within a dynamic equilibrium (fast exchange regime on the NMR chemical shift time scale) among numerous states in which only a small fraction is helical at any given instant in time. In any event, the presence of an alpha-helical conformation in the CaMK-R peptide is consistent with this type of structure in the R segment of the parent CaMKII (Figure 2B, magenta).

### 2.3 HSQC NMR reveals the CaMK-R-interacting surface on CRABP1

**FIGURE 3:**
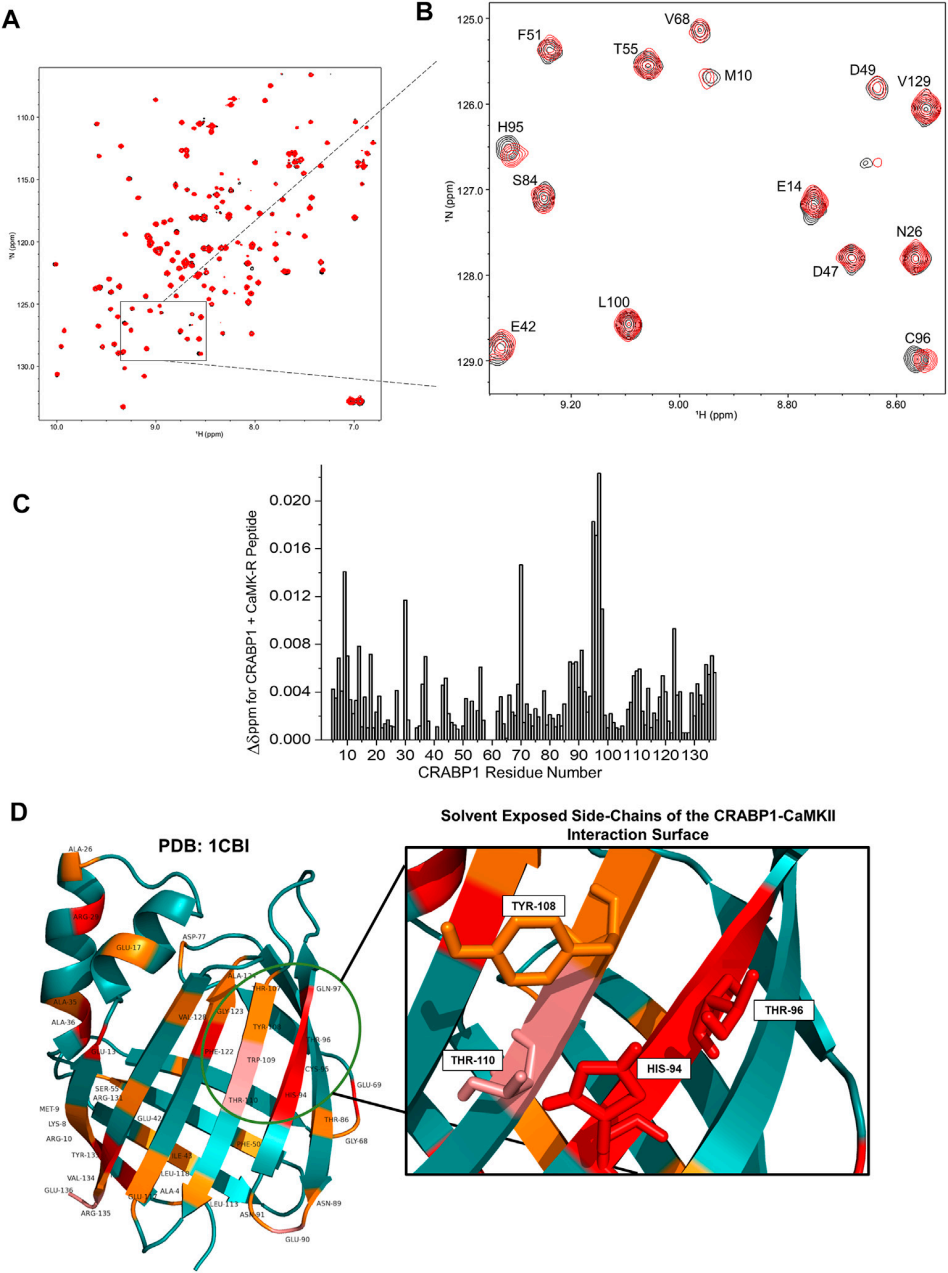
NMR reveals a CaMKII interaction surface on the beta-sheet face of CRABP1. **(A)** A full HSQC spectrum of CRABP1 alone (black, 19 uM) with a spectrum of CRABP1 (19 uM) + CaMK-R peptide (200 uM, red) overlaid. **(B)** Expanded region of the HSQC showing clear chemical shifts of CRABP1 residues upon CaMK-R addition. CRABP1 residue numbering is based on the complete 137-amino acid sequence, which includes the initiating methionine. HSQC spectra were generated using NMRFAM-Sparky. **(C)** Maximal chemical shift changes (∆δ) of CRABP1 residues resulting from CaMK-R addition plotted against the CRABP1 residue number. The plot was generated using Origin software. **(D)** CRABP1 residues with maximal chemical shift changes >2 standard deviations above (red), >1 standard deviation above (pink), or equal to the mean (orange) mapped to the CRABP1 crystal structure (PDB 1CBI). The green circle indicates the proposed CaMK-R binding site, and the inset shows solvent-exposed side-chains. CRABP1 residue numbering is based on the 136-amino acid sequence of the crystal structure of CRABP1 (PDB 1CB1), which lacks the initiating methionine. Images were generated using PyMOL Software.

To first assess whether CaMK-R interacts with CRABP1 in solution, we carried out ^15^N-^1^H HSQC experiments with ^15^N-labeled CRABP1 in the absence of CaMK-R (19 uM, black peaks) and the presence of CaMK-R (19 uM CRABP1 plus 200 uM CaMK-R, red peaks) (Figure 3A). As exemplified in the HSQC expansion shown in Figure 3B, it is apparent that some peaks are chemically shifted, indicating that the CaMKII peptide indeed interacts with CRABP1. Using resonance assignments for CRABP1 (Wook Park et al., 2019), we calculated chemical shift perturbations as plotted vs. the amino acid sequence of CRABP1 in Figure 3C. This chemical shift map identifies several CRABP1 residues that are significantly shifted, ranging from > 2SD from the mean (red highlight), >1SD (pink), equal to the mean (orange), and < 1SD (cyan). A complete list of CRABP1 residues with maximal chemical shifts is provided in Supplementary Table S1. These changes are highlighted on the crystal structure of CRABP1 (PDB: 1CBI) and identify a set of proximal residues within beta strands 7 and 8 as the likely CaMK-R-interacting surface (Figure 3D, green circle). Furthermore, residues His94, Thr96, Tyr108, and Thr110 are solvent-exposed, and, therefore, likely directly interact with CaMK-R. It should be noted that the residue numbering of these mutants is based on the sequence derived from the crystal structure of CRABP1 (PDB: 1CBI). Some chemically shifted resonances fall outside of this proposed interaction surface and are likely the result of allosteric effects resulting from the CRABP1–CaMK-R interaction. Taken together, these HSQC data indicate that CaMK-R interacts with CRABP1, at least at residues His94, Thr96, Tyr108, and Thr110. It should be noted that the CRABP1 residue numbering in Figure 3B is based on the complete 137-amino acid sequence, which includes the initiating methionine, whereas, in Figure 3D, the residue numbering is based on the 136-amino acid sequence of the CRABP1 protein used to determine the crystal structure, which lacks the initiating methionine. Henceforth, descriptions of the relevant CRABP1 residues in the subsequent studies described as follows are based on their numbering in the crystal structure shown in Figure 3D.

As mentioned previously, CaMK-R has some transient alpha-helical structures (Figure 2; Supplementary Figure S1). Interestingly, the regulatory segment of the CaMKII protein itself undergoes conformational changes that depend on the CaMKII activation state. In its autoinhibited form, the N-terminal portion of the regulatory segment indeed has a helical structure (Figure 2B, magenta). However, upon activation and exposure of Thr286/7 for autophosphorylation, the N-terminal portion loses its helical structure, whereas the C-terminal portion forms a helical structure (Rellos et al., 2010; Chao et al., 2011). The transient N-terminal helical structure in CaMK-R suggests that any *in situ* interaction would likely be in the context of the autoinhibited, inactive state of CaMKII.

### 2.4 Production and characterization of CRABP1 alanine mutant proteins for molecular studies

**FIGURE 4:**
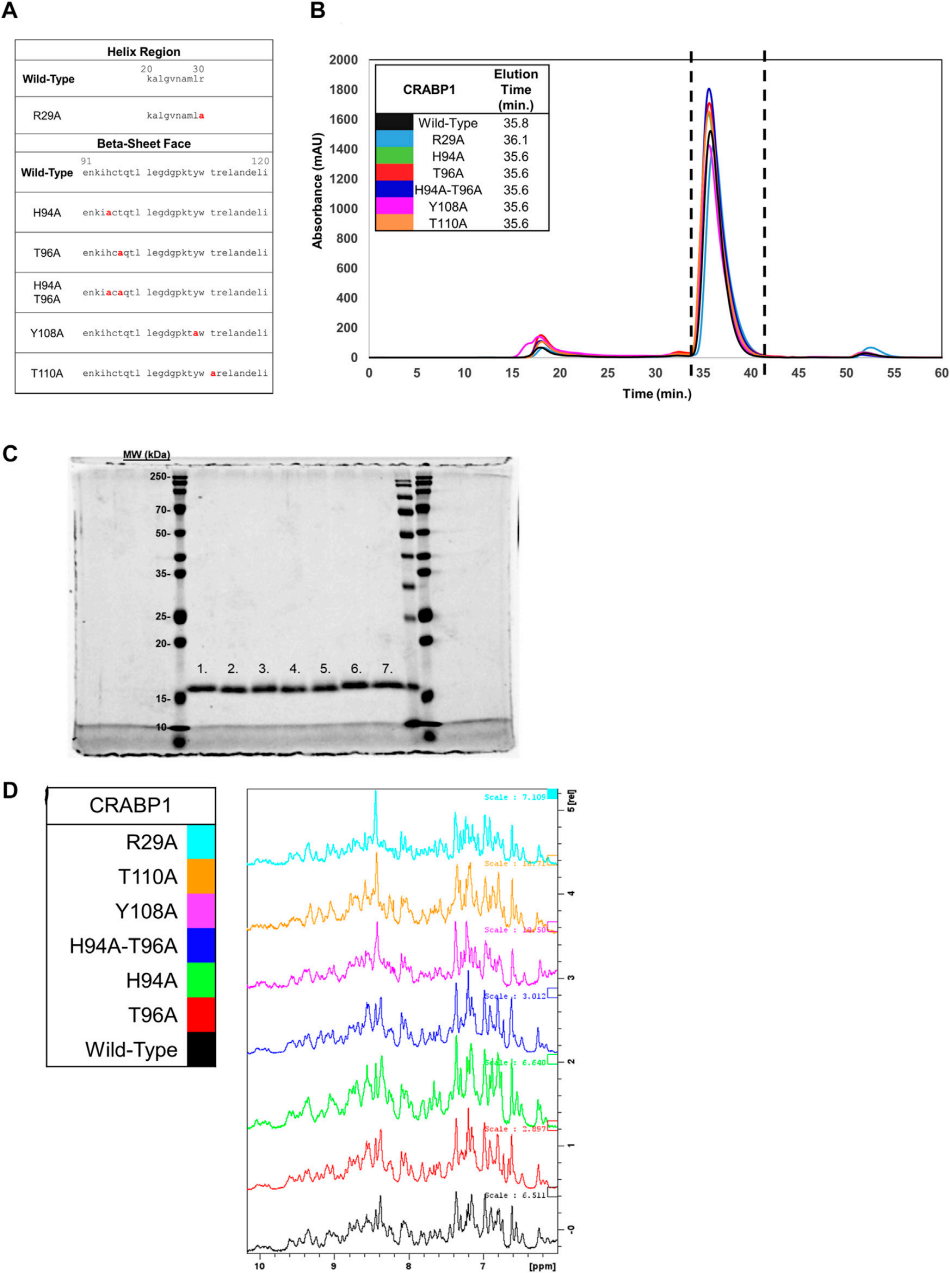
Protein fold is retained in CRABP1 mutants. **(A)** Sequence positions of CRABP1 alanine point mutations (red). **(B)** Size exclusion chromatography (SEC) profile and elution times of WT CRABP1 and alanine mutants collected during purification. The following masses of WT CRABP1 and alanine mutants were subjected to SEC: WT (5 mg), R29A (4.75 mg), H94A (5 mg), T96A (5.6 mg), H94A-T96A (5 mg), Y108A (4.65 mg), and T110A (5.4 mg). The dashed lines (---) indicate the relevant protein fractions collected for subsequent studies. SEC profiles and elution time data were monitored using the absorbance at 280 nm (mAU). The SEC profile was plotted using Microsoft Excel. **(C)** Coomassie-stained SDS-PAGE gel of purified, His-Tagged CRABP1 mutants (1 ug). Lane labels are as follows: wild-type (WT) (1), R29A (2), H94A (3), T96A (4), H94A-T96A double mutants (5), Y108A (6), and T110 (7). The first, second to the last, and last lanes show molecular weight markers from two different manufacturers. **(D)** 1D NMR spectra of the amide region of WT CRABP1 and mutants. Spectra scales were adjusted accordingly to visualize signal peaks. Spectra were generated using Bruker software.

Our proposed interaction surface between CRABP1 and CaMK-R (Figure 3D, green circle) was elucidated using HSQC NMR experiments. We then employed site-directed mutagenesis to produce alanine-substituted variants of the proposed interacting residues His94, Thr96, Tyr108, and Thr110. In addition, we generated an Arg29 mutant to assess whether chemical shifts of this residue are indeed the result of binding-induced allosteric effects (Figure 4A). Each alanine mutant was purified as a His-tagged recombinant protein isolated from *E. coli*. To determine if the introduction of these alanine point mutations altered the behavior of CRABP1, we monitored the size exclusion chromatography (SEC) profile during purification procedures and then performed SDS-PAGE profiling. When compared to WT CRABP1, these alanine mutants appear to behave similarly in solution. This is evident in their similar elution times and peak shapes (Figure 4B). The R29A mutant majorly appears to differ from WT CRABP1, with R29A having an elution time of 36.1 min, while WT CRABP1 has an elution time of 35.8 min (Figure 4B). The dashed lines (---) in Figure 4B indicate the protein fractions collected from the SEC procedures for SDS-PAGE profiling and molecular experiments. When subjected to SDS-PAGE, WT CRABP1 and alanine mutants also appear to have a similar migration pattern (Figure 4C). Therefore, in terms of in-solution behavior and electrophoretic migration, these alanine mutants appear to behave similarly to the WT CRABP1.

To ensure that each mutant was folded as in the wild-type, parent protein and to avoid time-consuming production of ^15^N-labeled proteins, we performed 1D ^1^H NMR experiments of these mutants to compare their spectrum values with the NMR spectrum of the parent protein. Because NMR spectra for the wild-type CRABP1 protein (black) and each mutant are essentially the same (as indicated by the presence of several defined peaks in the 7–10 ppm amide region (Figure 4D), we concluded that these mutants are folded as the wild-type, parent CRABP1 protein. Therefore, these alanine point mutants were verified as suitable for further molecular studies.

### 2.5 CRABP1 prefers interaction with inactive CaMKII

As mentioned previously, we have previously reported the interaction of CRABP1 with CaMKII, but whether and how this interaction may be affected by CaMKII’s activation status has not been determined. In order to address this issue, we employed an *in vitro* pull-down, protein interaction assay. His-tagged CRABP1 protein was purified from *E. coli* as the bait. To prepare both inactive and active forms of CaMKII (GFP-CaMKII, Addgene #21227) for pull-down reactions, we exploited mammalian (HEK293T) cells to express CaMKII and stimulated cells with ionomycin, which is known to robustly activate CaMKII (Liu and Hermann, 1978; Hudmon et al., 2005). The ionomycin-stimulated cells provide active CaMKII, whereas vehicle-treated cells provide inactive CaMKII.

**FIGURE 5:**
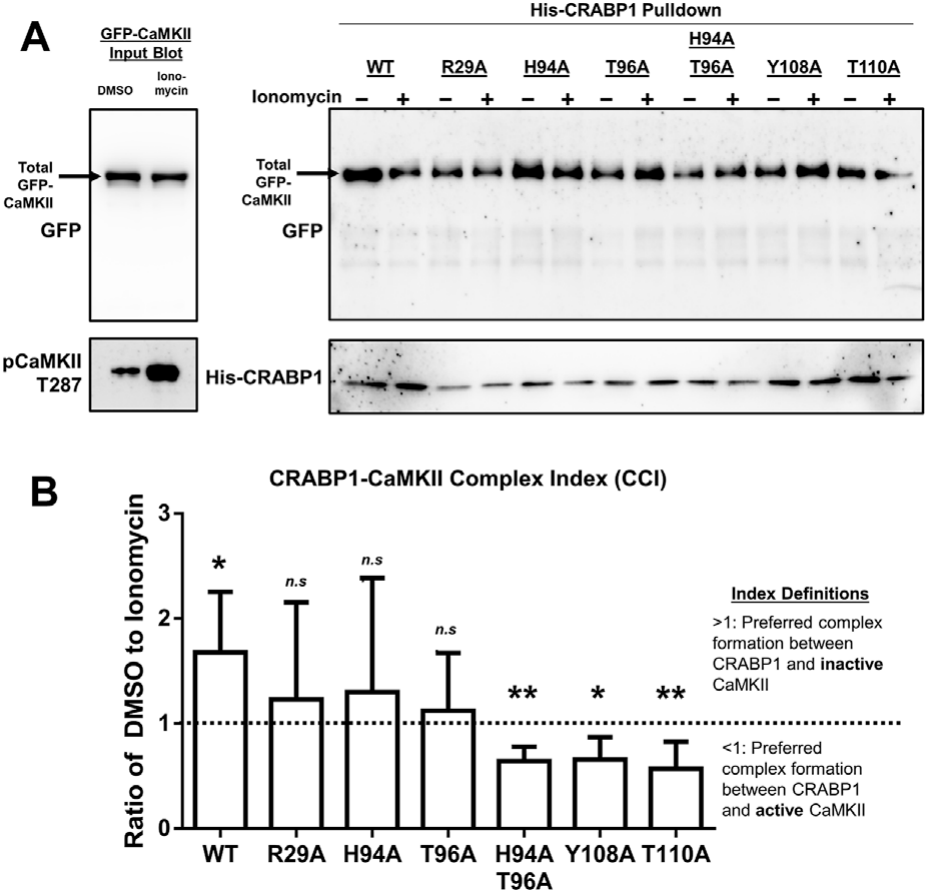
CRABP1 side-chain mutation dictates complex formation with CaMKII. **(A)** His-pull-down assay using purified, His-tagged wild-type (WT) CRABP1 or mutants (bait) and GFP-CaMKII (prey) lysate from HEK293T cells. Top left: input Western blot with a black arrow indicating the expected band positions for GFP-CaMKII, detected by anti-GFP antibody. Bottom left: Western blot to confirm that CaMKII is indeed activated upon ionomycin stimulation (10 uM, 10 min), marked by phosphorylated threonine 287 (pCaMKII T287) and detected by an anti-pCaMKII T287 antibody. Top right: His-pull-down assay of WT CRABP1 and mutants with unstimulated (–ionomycin) or stimulated (+ionomycin) lysate from the GFP-CaMKII lysate. The black arrow indicates the expected position of GFP-CaMKII in the pull-down assay, detected by the anti-GFP antibody. Bottom right: Western blot to detect the levels of WT and mutant CRABP1 present in each pull-down reaction, detected by the anti-His antibody. DMSO was used as a vehicle control (unstimulated condition) for ionomycin stimulation. **(B)** Quantification of the CRABP1–CaMKII Complex Index (CCI). The dashed line (---) marks 1 as the CCI index threshold. Values above the threshold of 1 (>1) indicate CRAPB1’s preference for inactive CaMKII. Values below the threshold (<1) indicate CRAPB1’s preference for active CaMKII. One-sample *t*-test was performed to compare if the CCI significantly differed from the threshold value of 1. *p ≤ 0.05, **p ≤ 0.01. “*n.s.*” indicates not significant. Error bars are presented as the mean ± standard deviation. (*n* = 5).

We used His-tagged wild-type and mutant (R29A, H94A, T96A, H94A-T96A double mutant, Y108A, and T110A) CRABP1, as well as GFP-CaMKII (GFP-CaMKII) prepared from HEK293T cells stimulated with vehicle control or ionomycin to perform a series of pull-down assays, monitored on Western blots. We first determined and confirmed the expected position of GFP-CaMKII from control and stimulated cells using an anti-GFP antibody, which detected both active and inactive CaMKII that migrated similarly (Figure 5A left panel, top arrow on the upper blot). Importantly, the anti-p-CaMKII T287 (active CaMKII) antibody detected robustly activated CaMKII from cells stimulated with ionomycin (Figure 5A, left panel, bottom blot). This result clearly demonstrated the success of active and inactive CaMKII preparations using this strategy. HEK293T cells transfected with the empty vector expressing only the GFP protein were used as a negative control (Supplementary Figure S3A).

With this strategy, experiments were carried out using the WT and mutated CRABP1 proteins as the bait to pull down inactive or active GFP-CaMKII, and the results are shown in Figure 5B. First, it is apparent that the WT CRABP1 was able to pull down much more inactive CaMKII (WT, – ionomycin) than active CaMKII (WT, + ionomycin), indicating that CRABP1 prefers to interact with the inactive CaMKII or that CRABP1 forms a more stable complex with the inactive CaMKII than its active form. The signals of inactive (– ionomycin) and active (+ionomycin) CaMKII bands were quantified, and the ratio of the inactive vs. active CaMKII signal was derived to obtain an arbitrary “CRABP1–CaMKII Complex Index” (CCI). A CCI of 1 would indicate the lack of preference for either active or inactive CaMKII, a CCI <1 would indicate preference for the active CaMKII, whereas a CCI >1 would indicate preference for the inactive CaMKII (see Section 4 for detailed quantification and calculation). The quantitated results obtained by performing multiple experiments are shown in Figure 5B. In the case of WT CRABP1, a CCI (inactive/active CaMKII ratio) of approximately 2 was derived, i.e., CRABP1 prefers interaction with inactive CaMKII.

Interestingly, these CCI analyses show that all the mutated CRABP1s were very different from the WT CRABP1, as shown in Figure 5. These CRABP1 mutants could be categorized into two groups: 1) no preference for inactive or active CaMKII (i.e., a CCI of approximately 1 such as R29A, H94A, and T96A) and 2) preferential interaction with active CaMKII (pCaMKII T287) (i.e., a CCI significantly <1) such as the H94A-T96A double mutant, Y108A, and T110A. The amount of WT or mutant CRABP1 was monitored for normalization (described in Section 4.9) (Figure 5A, right panel, bottom blot).

Altogether, these data show that CRABP1 can discriminate the inactive versus the active CaMKII. Specifically, the WT CRABP1 prefers to complex with the inactive CaMKII, or it forms a more stable complex with the inactive CaMKII. This would provide one mechanism underlying the observed “dampening” effect of CRABP1 in modulating CaMKII activation. Importantly, disrupting any of the residues identified from NMR data caused a loss of this discriminating effect, probably because these residues are all important, such that any alternation would compromise the unique conformation of the beta-sheet surface of CRABP1 (such as H94A, T96A, Y108A, and T110A mutants) or the allosteric helix region (the R29A mutant), both required for their ability to preferentially interact with the inactive CaMKII. Surprisingly, certain mutations, such as Y108A, T110A, and H94A-T96A, completely reversed CRABP1’s discriminatory property and preferred to form a complex with the active CaMKII (see Section 3).

### 2.6 Functional consequences of disrupting the CRABP1–CaMKII interaction

The CRABP1-mediated dampening of CaMKII activation was previously determined in an established mammalian culture system where HEK293T cells were co-transfected with CRABP1 and CaMKII, followed by monitoring the status of in-cell CaMKII activation (Lin et al., 2022). Using this established in-cell culture assay, we then determined the functional consequence of expressing WT and various mutated CRABP1 proteins also examined in protein interaction studies described previously in Figure 5.

**FIGURE 6:**
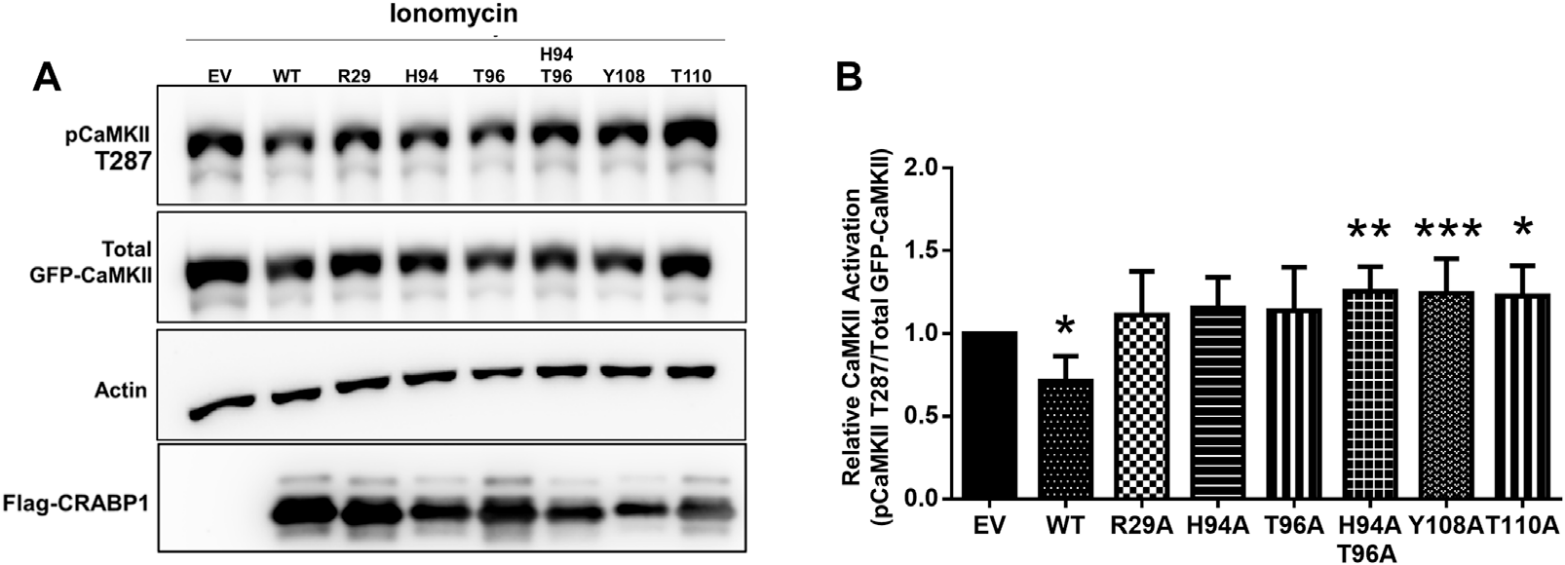
CRAPB1 side-chain mutation modulates dampening of CaMKII activation. **(A,B)** Western blot and quantification of the in-cell CRABP1–CaMKII assay under ionomycin stimulation (10 uM, 10 min). Flag-tagged wild-type (WT) CRABP1 or mutants were co-transfected with GFP-CaMKII beta in HEK293T cells. As a control empty vector (EV), the backbone was co-transfected with GFP-CaMKII beta. CaMKII activation was detected by a CaMKII phospho-threonine 287 (pCaMKII T287) antibody. Anti-GFP was used to detect total GFP-CaMKII expression. β-actin was used as a loading control. Anti-Flag was used to detect WT Flag-CRABP1 and mutant expression. One-way ANOVA, followed by Dunnett’s test for multiple comparisons, was performed to compare WT or mutant CRABP1 against EV control. *p ≤ 0.05, **p ≤ 0.01, ***p ≤ 0.001. Error bars are presented as the mean ± standard deviation. (*n* = 6).

As expected, WT CRABP1 significantly dampened CaMKII activation, indicated by a significant decrease of relative CaMKII activation (marked by reduced phospho-Thr287 and pCaMKII T287) (Figures 6A, B). The mutants that lost CaMKII-discriminating ability such as R29A (allosteric, helix position), H94A, and T96A (CaMKII binding site, beta-sheet face) failed to significantly affect CaMKII activation. Interestingly, H94A-T96A double mutant, Y108A, and T110A (all prefer interaction with the active CaMKII, Figure 5) increased CaMKII activation (Figures 6A, B). Together, the protein interaction (Figure 5) and functional (Figure 6) data indicate that disruption of an allosteric site (R29A) or certain residues within the CaMKII-binding sites (H94A and T96A) causes CRABP1 to lose its CaMKII-discriminating and -dampening effects, whereas certain mutations such as H94A-T96A, Y108A, and T110A can result in an opposing phenotype of CRABP1, i.e., they all prefer interaction with active CaMKII and increase CaMKII activation.

These data show that the ability of CRABP1 to discriminate between the inactive and active forms of CaMKII underlies its function in regulating CaMKII activation. The WT protein preferentially forms a complex with inactive CaMKII, probably stabilizing the inactive kinase, thereby dampening its activation. A disruption in the key CaMKII-interacting surface or the alpha-helix segment of CRABP1 would impact its ability to discriminate inactive from active forms of CaMKII, with corresponding changes in its functional effect with regard to CaMKII activation.

## 3 Discussion

**FIGURE 7:**
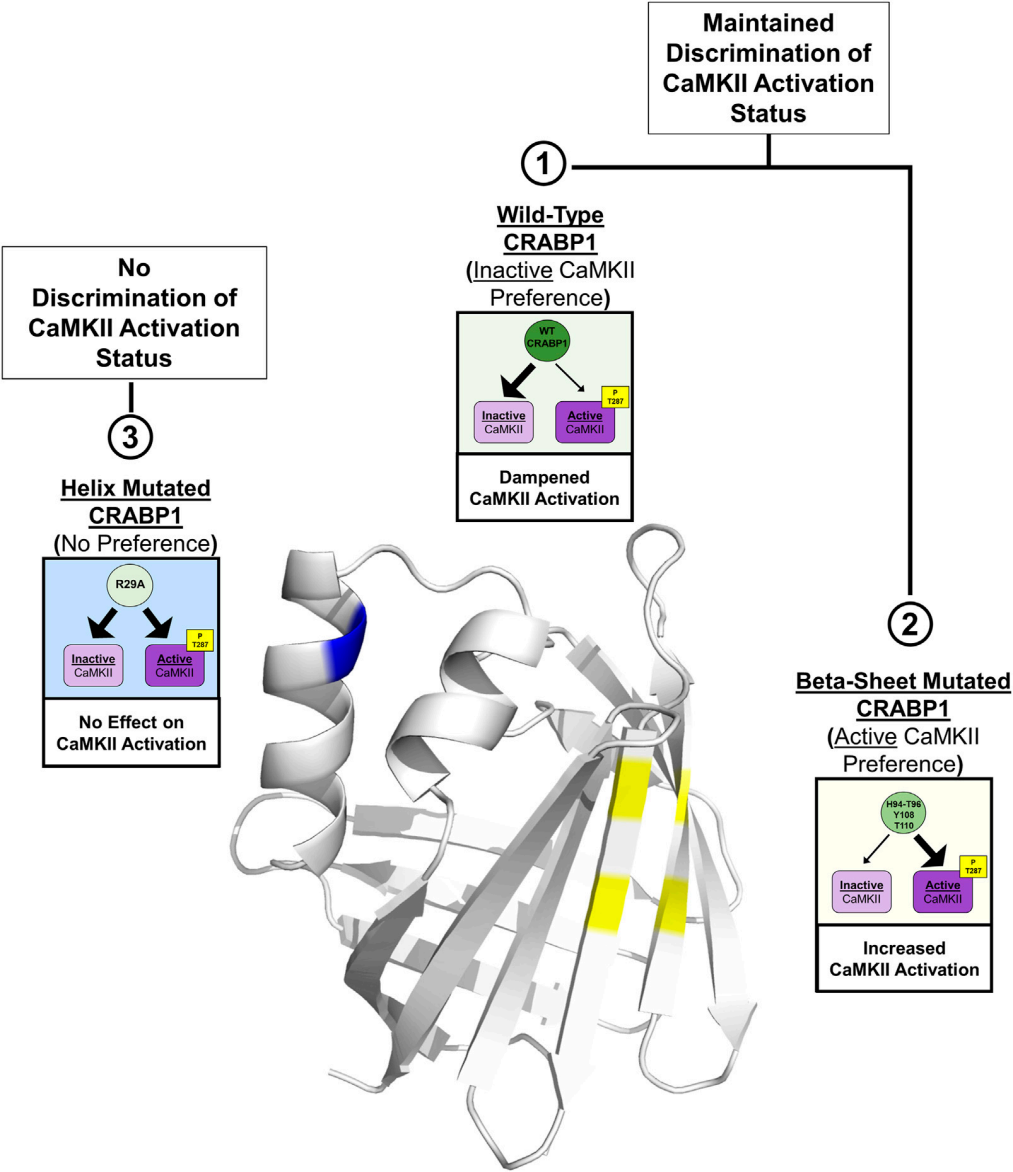
Structural model for CRABP1-mediated regulation of CaMKII. The crystal structure for Apo-CRABP1 (PDB 1CBI) with mutated sites on the helix motif (blue) and beta-sheet face (yellow) is indicated. Box 1 depicts the proposed dampening action of wild-type (WT) CRABP1. WT-CRABP1 has increased interaction with inactive CaMKII (large arrow), resulting in dampened CaMKII activation. Box 2 depicts the functional consequence of disruptive mutations on the proposed interaction surface on the beta-sheet face (H94A-T96A, Y108A, and T110A), which results in increased interaction (large arrow) with active CaMKII and subsequent increase in CaMKII activation, marked by T287 phosphorylation. Box 3 depicts mutation within the helix portion (blue), resulting in a loss of preference for either inactive or active CaMKII, resulting in no effect on CaMKII activation. Both WT CRABP1 and beta-sheet surface mutations maintain the ability to discriminate between inactive and active CaMKII (boxes 1 and 2) to modulate CaMKII activation, whereas mutations on the helix motif disrupt this discrimination, resulting in a loss of CRABP1-mediated CaMKII modulation (Box 3). This summary image was created using BioRender.com.

In this study, we identify an interaction surface on CRABP1 for CaMKII binding, along with an allosteric site located on the alpha-helix segment of CRABP1. *In vitro* interaction studies identify the ability of CRABP1 to discriminate between the inactive and active forms of CaMKII, with a preference toward association with the inactive CaMKII, which may underline CRABP1’s dampening effect in CaMKII activation. Disruptions of residues within the CaMKII-binding site on the beta-sheet barrel or the allosteric region within the alpha-helix cause a loss of this discriminatory ability or a shift in CRABP1 to preferentially associate with active CaMKII. Figure 7 depicts the proposed structural basis of CRABP1-mediated regulation of CaMKII. WT CRABP1 preferentially complexes with inactive CaMKII, resulting in dampened CaMKII activation (Box 1). Disruption of residues within the CaMKII-binding surface on the beta-sheet face (H94A-T96A double mutant, Y108A, and T110A) results in CRABP1 preferentially complexing with the active form of CaMKII, marked by Thr287 phosphorylation. This would cause enhanced kinase activation (Box 2). Disruption of allosteric residues (R29A) causes a loss of this discriminatory function of CRABP1 in association with CaMKII (Box 3).

This finding that CRABP1 can differentiate between inactive CaMKII and active CaMKII (marked by pThr286/7) is most interesting. The inactive and active forms of CaMKII are conformationally very distinct from each other (Bhattacharyya et al., 2020); our results would indicate that CRABP1 can structurally discern the inactive and active CaMKII conformations. To further validate these findings, it would be of most importance to examine the entire complex formation between CRABP1 and CaMKII in future studies by utilizing purified CRABP1 along with purified CaMKII in its active and inactive conformations. Additionally, the results suggest that CRABP1 residue side-chains are important in conferring this discriminatory ability (Figure 5). It is tempting to speculate that side-chain identity is not only important in the context of CaMKII regulation but also in a broader sense with regard to CRABP1’s function in RA’s non-canonical signaling pathways modulating multiple signaling pathways, such as MAPK versus CaMKII. To this end, it is interesting that the interaction surface on CRABP1 for Raf binding (Wook Park et al., 2019) spans strands 6 and 7, whereas the interaction surface for CaMKII identified in this study spans strands 7 and 8. Furthermore, the allosterically affected residues are different for CaMKII binding versus Raf binding. These interesting features would support the notion that stringent functional constraints on CRABP1, for its role in safeguarding multiple signaling pathways important for various physiological processes, would provide an evolutionary pressure to conserve its primary sequence across species and throughout evolution.

Finally, mutations drastically affecting side-chains within the CaMKII-interacting b-strand surface (H94A-T96A, Y108A, and T110A) revealed an especially interesting phenotype. It appears that the destruction of these side-chains can apparently transform CRABP1 into an activator of CaMKII, i.e., preferentially associate with active CaMKII. This would suggest that not only do these residues aid in maintaining CRABP1’s ability to differentiate among CaMKII conformations, but also the modulatory activity of CRABP1 could potentially be “tuned” toward a desired signaling outcome, such as by binding of various ligands. Our previous studies have reported that the binding of the endogenous CRABP1 ligand, RA, dramatically increases the thermal stability of CRABP1, clearly altering the structural dynamics of CRABP1 (Celej et al., 2003; Nhieu et al., 2023). Furthermore, synthetic CRABP1-binding ligands C3, C4, and C32 also increase the thermal stability, albeit with a more subtle magnitude. Nevertheless, these ligands could elicit CRABP1-dependent biological effects in cultured cell models of cancers and MN degeneration. The potentials of CRABP1 side-chains within the ligand-binding pocket, i.e., the beta-barrel, provide an exciting opportunity in future rationale designs of CRABP1 therapeutics.

## 4 Materials and methods

### 4.1 DNA constructs and chemicals

The GFP-CaMKII beta construct was obtained from Addgene (Addgene Cat #21227). The His-tagged, wild-type (WT) CRABP1 DNA construct for bacterial expression and subsequent protein purification was generated as described in Wook Park et al. (2019). The Flag-tagged, WT CRABP1 DNA constructs for mammalian expression used in HEK293T CRABP1-CaMKII cell assays were described in Wook Park et al. (2019). Constructs of alanine point mutants of His-tagged CRABP1 and Flag-tagged CRABP1 were generated using the Q5^®^ Site-Directed Mutagenesis Kit (New England BioLabs Inc., Cat #E0554S) according to the manufacturer’s instructions. To validate successful site-directed mutagenesis, the relevant regions of the CRABP1 insert from each mutant construct were validated by Sanger sequencing, which was performed by the University of Minnesota Genomics Center Facility (Minneapolis, MN).

Chemical reagents utilized in this study are as follows: Tris-d11 solution (Sigma Cat # 486248), sodium acetate-d3 (Sigma Cat # 176079), dithiothreitol (DTT) (Gold Biotechnology Cat# DTT10), Tris (2-carboxyethyl)phosphine hydrochloride solution (Sigma Cat # 646547), dimethyl sulfoxide (DMSO) (Sigma Cat #D8418), and ionomycin salt (Sigma Cat #I0634). Ionomycin for molecular and cell studies was prepared by dissolving it in DMSO.

### 4.2 Cell culture

HEK293T cells (ATCC) were maintained as described in Nhieu et al. (2023). Briefly, HEK293T cells were maintained in DMEM (Thermo Fisher Cat # 11965092) supplemented with 10% FBS and 1% penicillin–streptomycin (Thermo Fisher Cat # 15140122) in an incubator maintained at 37°C and 5% CO_2_. HEK293T cells were routinely tested for and found to be negative for *mycoplasma*.

### 4.3 Protein expression and purification of WT CRABP1, alanine mutants, and ^15^ N WT CRABP1

Protein expression was carried out as described in Wook Park et al. (2019), with the following modification of growing the induced culture at 16°C overnight. The bacterial pellet was isolated via centrifugation (×5,000 g, 15 min, 4°C) and resuspended in lysis buffer (1 x PBS, 10 mM imidazole, pH 8.0). Lysis was carried out by three rapid freeze–thaw cycles utilizing liquid nitrogen and a water bath warmed to 55°C, followed by five rounds of sonication on ice for 90 s with a 2-s pulse. A 90-s resting period for cooling was conducted in between each round of sonication. The bacterial lysate was subjected to high-speed centrifugation (23,000 x g, 60 min, 4°C) to clear the lysate of cell debris and/or aggregated protein. The lysate was then filtered through a 0.25-um filter (Cytvia Cat # 4,652) for further clarification of debris. Additional imidazole was added to a final concentration of 20 mM to the lysate to prevent non-specific interactions with the affinity nickel column resin. The lysate was run through the nickel resin column (HisTrap FF, Cytvia Cat #17531901) twice for optimal binding to the resin. The HisTrap column was then washed with 25 column volumes (CV) of lysis buffer. The His-tagged protein was eluted with 12.5 CVs of elution buffer (1 x PBS, 500 mM imidazole, pH 8.0). Dithiothreitol (DTT) was added to a final concentration of 1 mM to the eluted protein to ensure cysteine reduction. The eluted protein was then concentrated using an Amicon spin filter with a molecular weight cut-off of 10 kDa (MilliporeSigma Cat # UFC9010) for downstream size exclusion chromatography (SEC). SEC was performed using an AKTA FPLC system with a Frac 950 fraction collector controlled by Unicorn version 5.31 software. SEC was performed with a Superdex 200 increase 10/300 gl column (Cytvia Cat # 28990944) in 1 X PBS, pH 8.0. The following masses of WT CRABP1 and alanine mutants were subjected to SEC profiling during purification procedures: WT (5 mg), R29A (4.75 mg), H94A (5 mg), T96A (5.6 mg), H94A-T96A (5 mg), Y108A (4.65 mg), and T110A (5.4 mg). Absorbance at 280 nm (A_280_) was used to monitor protein elution. Relevant fractions were collected and pooled and DTT was added to a final concentration of 1 mM. Protein concentration was measured using the absorbance at 280 nm (A_280_) on a NanoDrop machine (Thermo Fisher). For accurate quantification of the protein concentration, the extinction coefficient and expected molecular weight (MW) for WT and each CRABP1 mutant were determined using the Expasy ProtoParam tool (https://web.expasy.org/protparam/). The extinction coefficients are as follows: WT CRABP1, R29A, H94A, T96A, H94A-T96A, T110 20,970 M^−1^ cm^−1^, and Y108A 19,480 M^−1^ cm^−1^. WT CRABP1 mutants have an expected MW of approximately 17 kDa. ^15^N-labeled WT CRABP1 used in NMR experiments was expressed and purified as described in Park et al. (2019). The SDS-PAGE and SEC profiles of purified ^15^N-labeled WT CRABP1 used in NMR experiments are shown in Supplementary Figure S4. SEC and SDS-PAGE procedures for ^15^N-labeled CRABP1 were performed as described previously. A protein mass of 354 ug of ^15^N-labeled CRABP1 was subjected to SEC profiling.

Purity was assessed by running 1 ug of WT CRAPB1 and mutants on a 13.8% SDS polyacrylamide (v/v) gel and stained with Coomassie Brilliant Blue R-250 Staining Solution (Bio-Rad Cat # #1610436). Given the inherent variability across manufacturers, molecular weight markers from two different manufacturers (Prometheus Protein Biology Products Ca t# 83–650 and Thermo Fisher Cat # 26619) were used to assess the apparent molecular weight of WT and CRABP1 mutants. The protein gel was then de-stained by boiling with ddh_2_0. Images were acquired using the Bio-Rad Chemi Doc Imager.

### 4.4 CaMK-R peptide synthesis and preparation for NMR experiments

The CaMK-R peptide used in NMR experiments was synthesized by the Department of Biochemistry, Biophysics, and Molecular Biology Peptide Synthesis Services at the University of Minnesota. For NMR experiments, the CaMK-R peptide was prepared by resuspending the lyophilized peptide in NMR buffer (30 mM d11-Tris-d3-acetate, pH 6.2, 75 mM Na_2_SO_4_, 10 µM ZnCl_2_, and 1 mM TCEP). The peptide concentration was measured using the absorbance at 205 nm with an extinction coefficient of 31 mL mg^−1^ cm^−1^ (Anthis and Clore, 2013) on a NanoDrop machine.

### 4.5 NMR sample preparation, experimental parameters, and data analysis

All NMR experiments were performed using samples made up as follows: 30 mM d11-Tris-d3-acetate, pH 6.2, 75 mM Na_2_SO_4_, 10 µM ZnCl_2_, 1 mM TCEP, made up using a 95% H_2_O/5% D_2_O mixture (v/v). All NMR experiments were carried out at 30°C on a Bruker 850 or 900 MHz Avance III NMR spectrometer equipped with an H/C/N triple-resonance probe and x/y/z triple-axis pulse field gradient unit. One-dimensional experiments with excitation sculpting for solvent suppression were carried out on parent CRABP1 (85 uM), mutant proteins (50–210 uM), and CAMKII-R (100 uM) using the following parameters: 16 ppm sweep width, 32K data points, and 32 scans. A 950 uM CAMKII-R sample was used to collect a gradient sensitivity-enhanced version of the two-dimensional 1H-15N (natural abundance) HSQC experiment (1,024 scans) with 256 (t1) x 2048 (t2) complex data points and 32 ppm and 1 ppm sweep width in the 15N and 1H dimensions, respectively. Phase-sensitive versions of 2D NOESY (with WATERGATE solvent suppression) and TOCSY (with presaturation using MLEV) were collected on 100 uM CAMKII-R, using the following parameters: 256 (t1) x 2048 (t2) complex data points, 11 ppm sweep width, and 32 scans. Additionally, a 2D 1H-13C (natural abundance) HSQC with 512 (t1) x 2048 (t2) data points and 90 ppm and 16 ppm sweep width in the 13 C and 1H dimensions, respectively, was collected on the 100 uM CAMKII-R sample. Raw data were converted and processed using NMRPipe (Delaglio et al., 1995) and analyzed with NMRview (Johnson and Blevins, 1994).

### 4.6 *In vitro* His-pull-down assay

For *in vitro* pull-down studies, purified His-CRABP1 (WT or mutants) served as the bait protein to pull down the prey protein, GFP-CaMKII. The His-CRABP1 bait protein was prepared by binding purified His-CRABP1 (5 uM) to nickel-nitrilotriacetic acid resin (Ni-NTA, Qiagen) in a total volume of 500 ul of reaction buffer (50 mM Tris pH 8.0, 150 mM NaCl, 0.2% (v/v) NP-40, 20% glycerol, and 200 mM imidazole) for 1 h at 4°C with agitation.

To prepare HEK293T cells as a source of GFP-CaMKII for pull-down experiments, cells first were seeded at a density of 3 × 10^6^ cells into a 10-cm dish 18–24 h prior to transfection. On the day of transfection, cells were exchanged into incomplete DMEM (Thermo Fisher Cat # 11965092) and transfected with 10 ug of GFP-CaMKII DNA using polyethylenimine (PEI) max transfection reagent (Polysciences Cat # 24765). A PEI: DNA ratio of 1:3 was used (Boussif et al., 1995). Cells were then subjected to downstream pull-down assay experiments 48 h post-transfection.

HEK293T cells transfected with the empty backbone that expresses only GFP protein were used as a negative control. In order to generate inactive CaMKII prey protein and active CaMKII prey protein, HEK293T cells were treated with DMSO (vehicle control) or ionomycin (10 uM, 10 min), respectively. Treated cells were immediately harvested for whole-cell lysate protein extraction. Whole-cell lysate protein extraction was carried out by resuspending pelleted cells in lysis buffer (50 mM Tris pH 8.0, 150 mM NaCl, 0.2% (v/v) NP-40, 20% glycerol, and 1X protease–phosphatase inhibitor solution (Cell Signaling Cat # 58725)). Then, the cell lysate was centrifuged at high speed (16,000 x g, 15 min, 4°C) to remove debris. Then, the cell lysate protein extract was quantified using Bradford assay with Bradford reagent (Bio-Rad Cat # 5000001) on a Bio-Rad Smart Spec spectrometer.

Furthermore, 250 ug of GFP-CaMKII lysate (DMSO or ionomycin treated) was pre-cleared with incubation with Ni-NTA beads alone for 1 h at 4°C with agitation. The pre-cleared lysate was then incubated with bait His-CRABP1 (WT or mutants) in reaction buffer (50 mM Tris pH 8.0, 150 mM NaCl, 0.2% (v/v) NP-40, 20% glycerol, 200 mM imidazole, and 1X protease–phosphatase inhibitor solution) overnight with agitation at 4°C. Then, the beads from the pull-down reactions were washed for 30 s with agitation, five times with wash buffer (50 mM Tris pH 8.0, 150 mM NaCl, 0.2% (v/v) NP-40, 20% glycerol, and 20 mM imidazole). The reaction was terminated by removing the wash buffer and resuspending the reaction Ni-NTA beads in SDS lysis buffer (9 parts: 128 mM Tris base, 10% (v/v) glycerol, 4% (w/v) SDS, 0.1% (w/v) bromophenol blue, pH to 6.8 and 1 part: beta-mercaptoethanol). Then, a Western blot was performed as a readout for the pull-down assay. Anti-pCaMKII Thr286/7 was used to confirm that ionomycin stimulation indeed induced CaMKII activation. Anti-GFP was used to detect total pulled-down GFP-CaMKII, and anti-His was used to detect CRABP1 in the pull-down assay. All experiments were repeated at least three independent times (*n* = 5).

### 4.7 In-cell CRABP1–CaMKII assay

For in-cell CRABP1–CaMKII assays, HEK293T cells were seeded at a density of 2 × 10^5^ cells into each well of a 6-well plate a day prior to transfection. The PEI transfection reagent was used as described in Section 4.6. A total of 2.5 ug of GFP-CaMKII and CRABP1 (WT or mutant) was co-transfected with a 1:5 ratio of DNA mass (ug) of GFP-CaMKII to CRABP1. After 48 h post-transfection, cells were then subjected to downstream in-cell CRABP1–CaMKII assays. To stimulate CaMKII activation, HEK293T cells were treated with either DMSO (vehicle control) or ionomycin (5–10 min, 10 uM). Preliminary studies determined that 5–10 min was the optimal window for stimulation to consistently detect WT CRABP1 dampening of CaMKII and mutant effects on CaMKII activation. Cells co-transfected with an empty vector backbone and GFP-CaMKII were used as a control. Cells were then immediately harvested using SDS lysis buffer and subjected to downstream Western blot procedures. Anti-pCaMKII Thr286/7 was used to detect CaMKII activation, anti-GFP was used to detect total GFP-CaMKII, anti-β-actin was used to detect actin as a loading control, and anti-Flag was used to detect Flag-WT or mutant CRABP1. Experiments were repeated at least three independent times (*n* = 6).

### 4.8 Western blot

For in-cell CRABP1–CaMKII assays, cell lysates were separated on 9% (v/v) SDS polyacrylamide gels and transferred onto 0.45-µm PVDF membranes (Millipore Sigma Cat. IPVH00010). For His pull-down assays, reactions were separated on 10% (v/v) SDS polyacrylamide gels and transferred on a 0.45-µm PVDF membrane. Primary antibodies and their dilutions used include anti-p-CaMKII (cat #: 127,165, 1/1,000) from Cell Signaling-Danvers, MA, United States, anti-GFP (cat #: SC-9996, 1/1,000) from Santa Cruz Biotechnology, anti-β-Actin (cat #: SC-47778, 1/1,000) from Santa Cruz Biotechnology-Dallas, TX, United States, anti-FLAG from Sigma (cat#: F3165, 1:1,000), and anti-His (Cat # sc-8036, 1:1,000) from Santa Cruz Biotechnology-Dallas, TX, United States, and secondary antibodies used include goat anti-mouse-IgG-HRP (cat #: GTX26789, 1/5,000) from GeneTex, Irvine, CA, United States and goat anti-rabbit-IgG (cat #: 11–035-144, 1/2000) from Jackson ImmunoResearch, Ely, United Kingdom.

WesternBright ECL substrate was used for chemiluminescent detection of Western blot signals (Advansta Cat # K-12045-D50) A Bio-Rad ChemiDoc Imager, Hercules, CA, United States (cat #: 17001402) was used to collect images.

### 4.9 Data analysis and software

In order to clearly convey this discriminatory ability of CRABP1 and the functional consequences of CRABP1 mutation in pull-down assay experiments, we generated a numerical index called the “CRABP1–CaMKII Complex Index (CCI)”. To calculate this CCI, we first performed a densitometry analysis of Western blots to normalize GFP-CaMKII to His-CRABP1 in unstimulated (–ionomycin) and stimulated (+ionomycin) conditions. This calculation determines the relative amount of CRABP1 complexed with inactive (CRABP1–inactive CaMKII) or active CaMKII (CRABP1–active CaMKII). Then, the ratio of CRABP1–inactive CaMKII to CRABP1–active CaMKII (CRAPB1-inactive: CRABP1-active) was calculated. Theoretically, as this ratio approaches 1, the amount of inactive and active CaMKII bound to CRABP1 is equivalent. This would indicate a loss of the discriminatory function of CRABP1 to bind either inactive or active CaMKII. Thus, we set 1 as a threshold value to measure this discriminatory ability of CRABP1. If this ratio is greater than 1 (>1), this indicates that CRABP1 prefers inactive CaMKII. If this ratio is less than 1 (<1), this indicates that CRABP1 prefers active CaMKII. To quantify this CCI, we performed a one-sample *t*-test to determine if WT or CRABP1 mutants significantly differed from this theoretical threshold value of 1 (Student, 1908; Canavos, 1988). Pull-down experiments were performed at least three independent times (*n* = 5).

For in-cell CRABP1–CaMKII assay experiments, densitometric analysis of Western blots was performed by first normalizing pCaMKII T287 signal to total GFP-CaMKII for empty vector (EV) control, WT CRABP1, and mutant conditions. Then, the fold-change of empty vector (EV) (WT or mutant condition divided by EV control) was taken to calculate relative CaMKII activation. One-way ANOVA followed by Dunnett’s test for multiple comparisons (Dunnett, 1955) was performed to determine if WT or mutant CRABP1 differed from empty vector control. Pull-down experiments were performed at least three independent times (*n* = 6). Densitometric analysis was performed using Fiji ImageJ (Schindelin et al., 2012).

Software used in this study includes the following: PyMOL ver. 1.8.6.2 (Schrödinger, LLC; http://www.pymol.org/pymol) for rendering and creating figures for CRABP1 and CaMKII crystal structures; Bruker TopSpin ver. 3.5 or NFRAM-Sparky (Lee et al., 2015) was used to create spectra of NMR data; Origin software was used to plot chemical shifts NMR data; Unicorn version 5.31 was used for AKTA FPLC operation, SEC profile, and elution time monitoring; Microsoft Excel was used to plot SEC profiles; GraphPad Prism 6 was used for statistical analyses and graphs for pull-down and in-cell CRABP1–CaMKII assay experiments; Biorender.com was used to create the summary figure.

## Data Availability

The original contributions presented in the study are included in the article/Supplementary Material; further inquiries can be directed to the corresponding author.
