# Supplementary material for: Molecular basis for cellular retinoic acid-binding protein 1 in modulating CaMKII activation
Source: Front Mol Biosci. 2023 Sep 26;10:1268843. doi: 10.3389/fmolb.2023.1268843 (PMC10562560; doi:10.3389/fmolb.2023.1268843)
Supplement: Supplementary file 1 [file DataSheet1.docx]

Supplementary Material


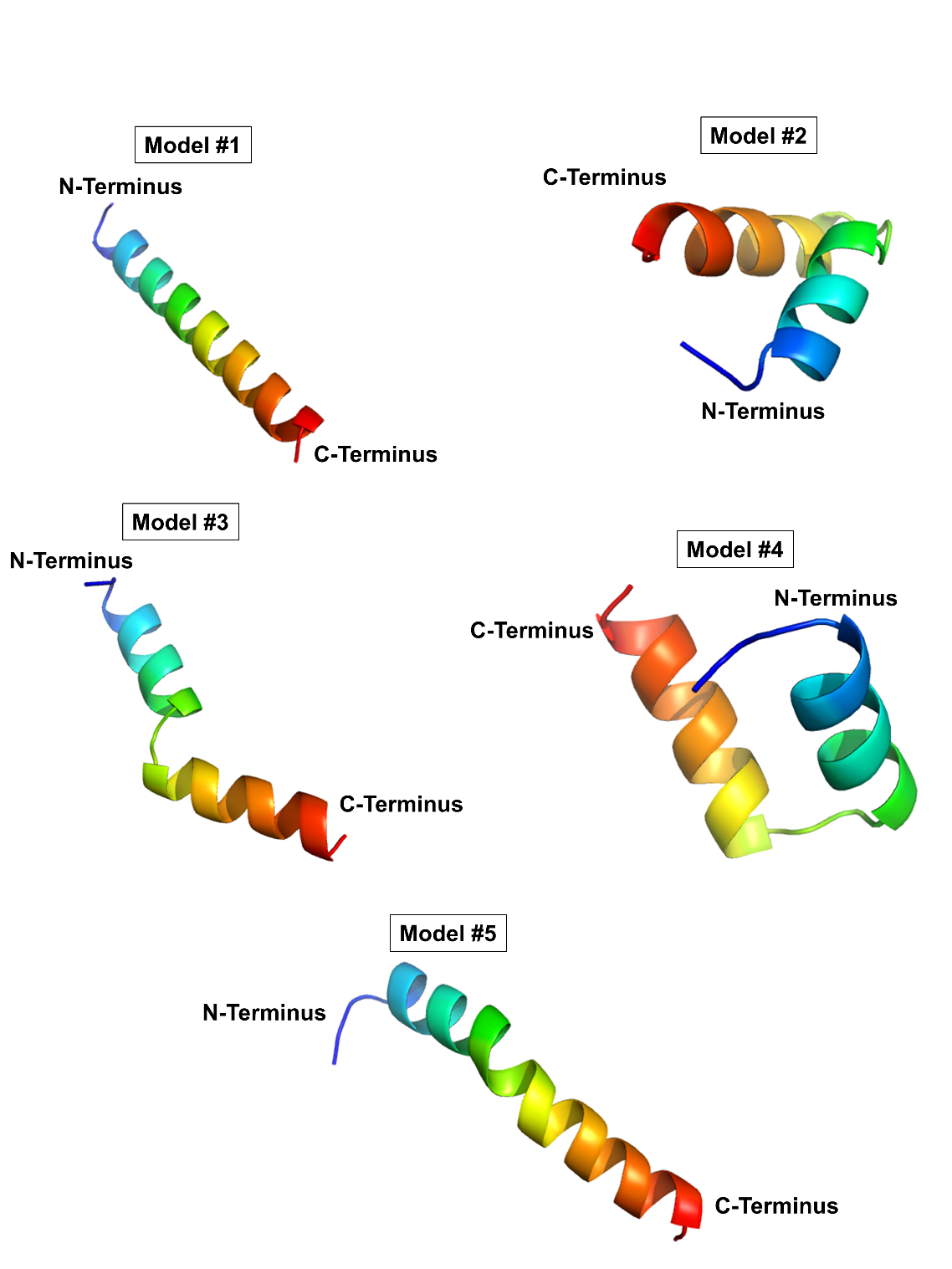


**Supplementary Figure 1.** Predicted secondary structure models for CaMK-R peptide. a) The top five predicted structures for the CaMK-R peptide computationally predicted using the PEP-FOLD Peptide Structure Prediction Server (<https://bioserv.rpbs.univ-paris-diderot.fr/services/PEP-FOLD/>)


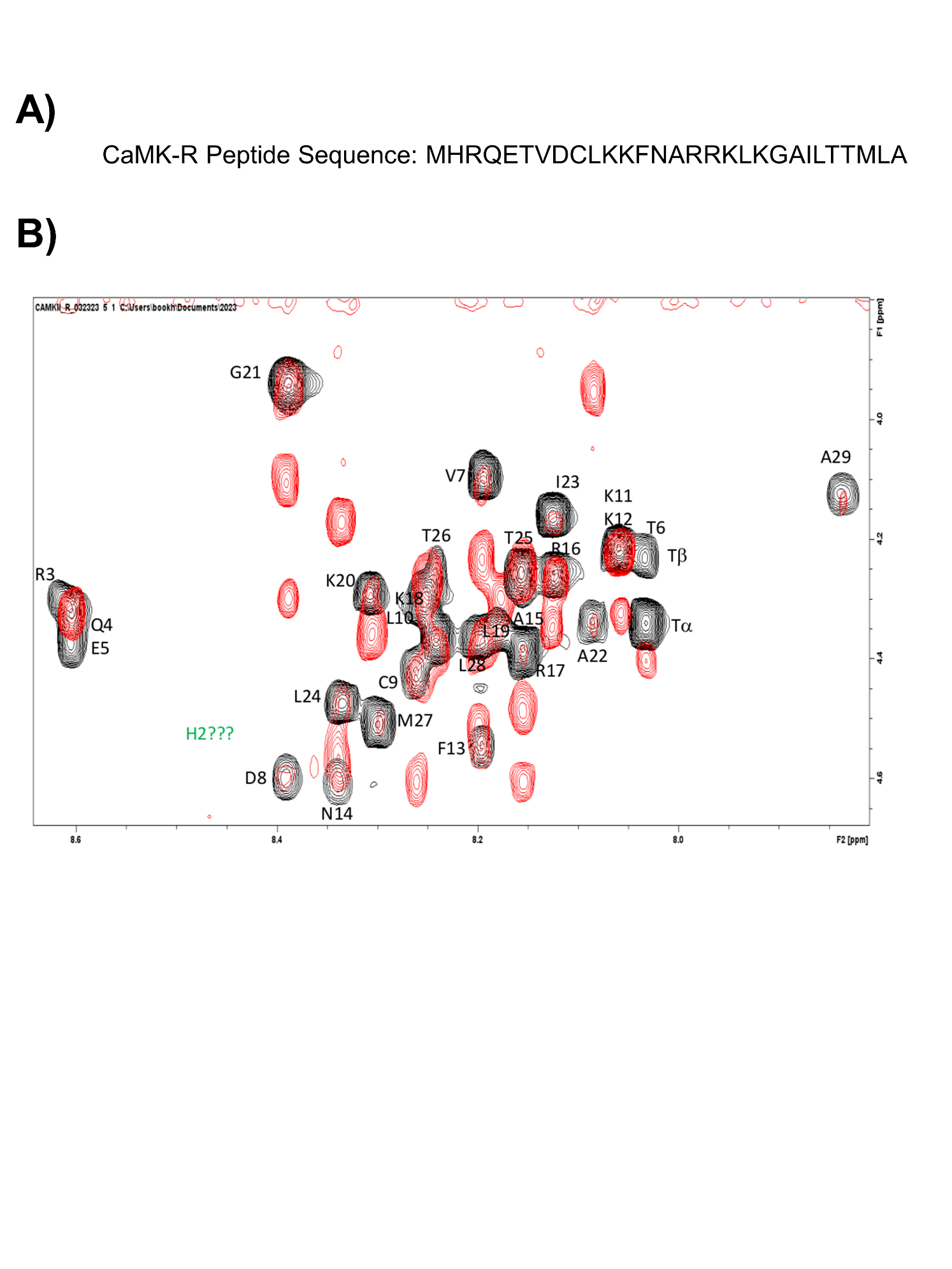


**Supplementary Figure 2.** TOCSY-NOESY assignments for CaMK-R peptide. a) The CaMK-R peptide sequence. b) Overlay of NOESY (red) spectra and TOCSY (black) spectra of the CaMK-R peptide used for CaMK-R assignment. Spectra were generated using Bruker software.


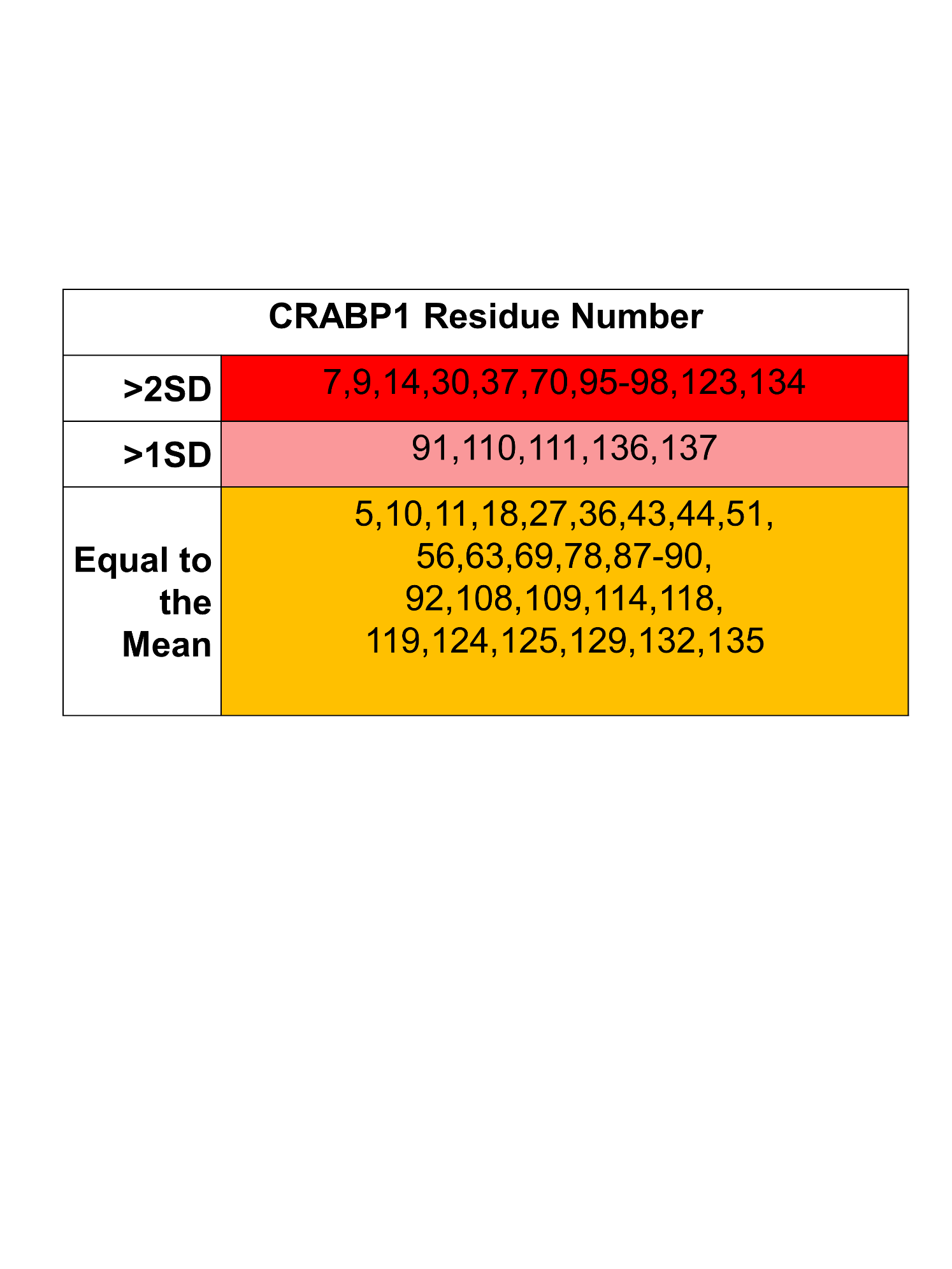


**Supplementary Table 1.** Maximal chemical shift changes of CRABP1 residues in the presence of CaMK-R.


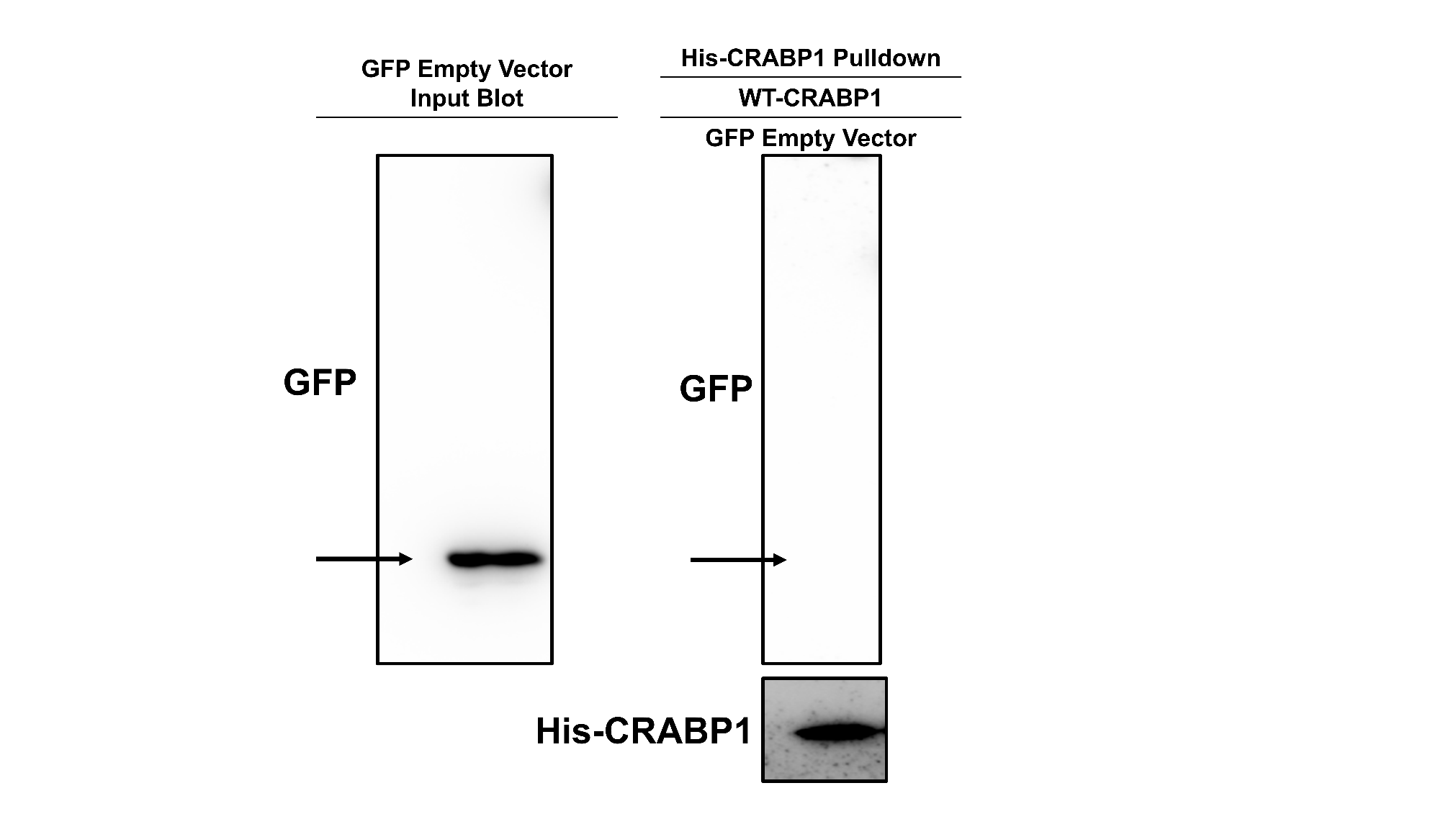


**Supplementary Figure 3.** Negative control reaction for His pull-down assay. Western blot of a negative control reaction for His pull-down using WT His-CRABP1 (bait) and GFP protein cell lysate. Left: Input blot for the expected position of GFP protein indicated by a black arrow. Right: His pulldown assay using wild-type His-CRABP1 and GFP protein lysate. Anti-GFP antibody was used to detect GFP protein, anti-His antibody was used to detect His-CRABP1. (n=5)


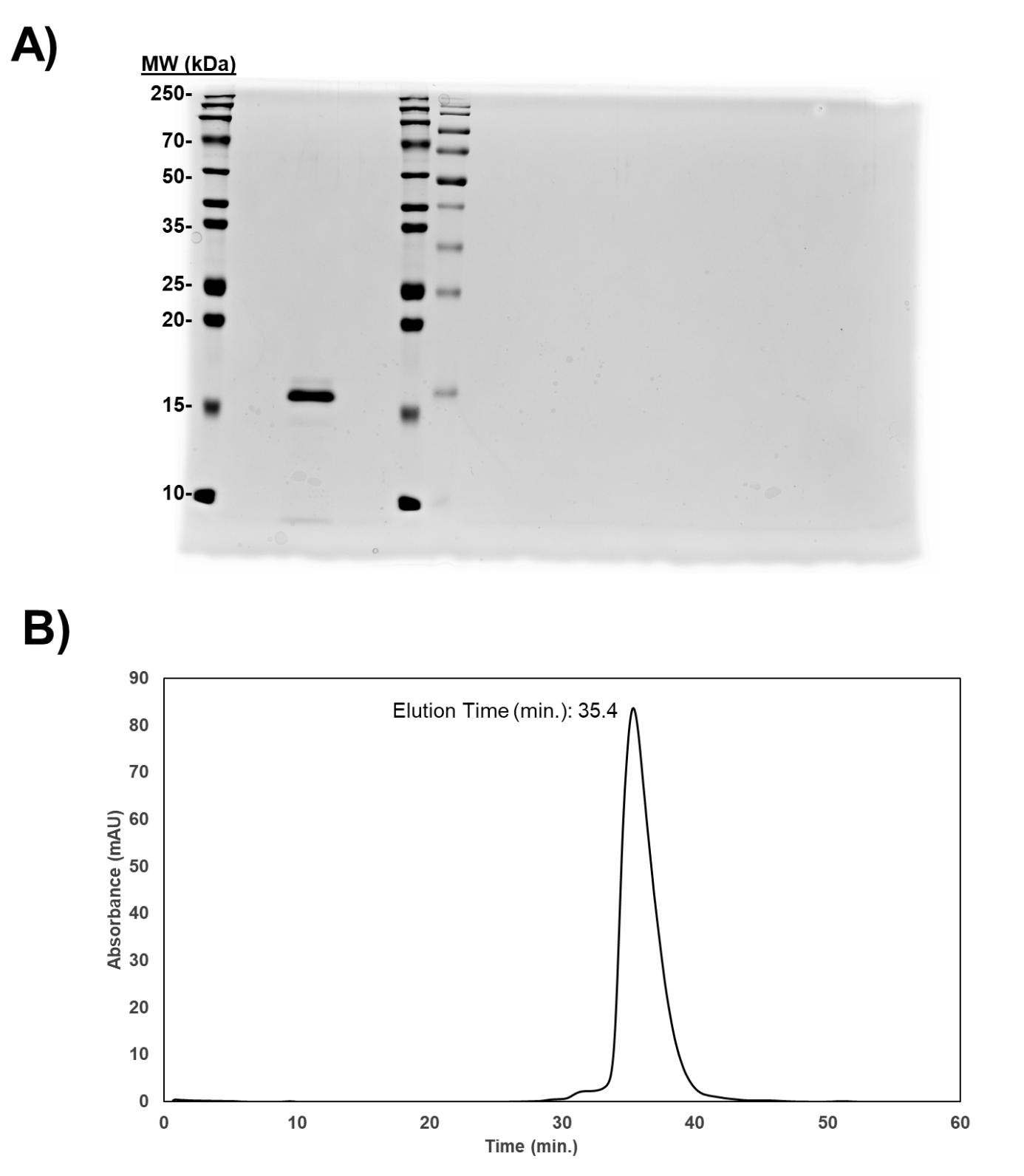


**Supplementary Figure 4.** SDS-PAGE and size exclusion profile of purified, ^15^N-labelled CRABP1. A) Coomassie stained, SDS-PAGE gel of ^15^N-labelled CRABP1 (1 ug). The last lane contains a molecular weight marker from a different manufacturer. B) Size exclusion profile of purified, ^15^N-labelled CRABP1 (354 ug). Absorbance at 280 nm (mAU) was monitored to detect the elution of ^15^N-labelled CRABP1 which occurred at a timepoint of 35.4 minutes. For detailed chromatography procedures see Methods section 4.3. Microsoft Excel was used to plot the chromatogram.
